# An Effective Non-Rigid Registration Approach for Ultrasound Images Based on the Improved Variational Model of Intensity, Local Phase Information and Descriptor Matching

**DOI:** 10.3390/jimaging12040156

**Published:** 2026-04-03

**Authors:** Kun Zhang, Jinming Xing, Qingtai Xiao

**Affiliations:** 1 Yunnan Bureau of Hydrology and Water Resources, Kunming 650106, China; 2 Yunnan Key Laboratory of Water Security, Kunming 650021, China; 3 Yunnan Water Investment Information Technology Co., Ltd., Kunming 650200, China; 4 School of Metallurgical and Energy Engineering, Kunming University of Science and Technology, Kunming 650093, China

**Keywords:** non-rigid registration, ultrasound image, local phase, descriptor matching, variational framework, warping technique

## Abstract

Ultrasound images have some limitations, such as low signal-to-noise ratio (SNR), speckle noise, lower dynamic range, blurred boundaries, and shadowing; therefore, ultrasound image registration is an important task for estimating tissue motion and analyzing tissue mechanical properties. In this paper, an effective non-rigid ultrasound image registration method is proposed. By integrating intensity, local phase information, and descriptor matching under a variational framework, we can find and track the non-rigid transformation of each pixel under diffeomorphism between the source and target images based on the warping technique. Experiments using simulation and in vivo ultrasound images of the human carotid artery are conducted to demonstrate the advantages of the proposed algorithm, which will act as an important supplement to current ultrasound image registration.

## 1. Introduction

Ultrasound imaging has played an important role in medical diagnosis and assessment in the past few decades because of its real-time, non-invasive, safety, and lower cost compared to other medical imaging modalities [1]. In medical applications, registration of ultrasound images can be used in compounding spatial images to improve image quality and contrast, estimating tissue motion, or analyzing tissue mechanical properties to deliver a treatment and monitoring lesion development to assist in making a surgery plan [2,3,4]. However, ultrasound images still have some limitations, such as low SNR, speckle noise, lower dynamic range, blurred boundaries, and shadowing, so ultrasound images make registration more challenging. Generally speaking, the non-rigid registration is more suitable for medical images than the rigid registration because of the physical characteristics of soft tissue changes in organs or tissues [5,6]. The optical flow algorithm is very popular in non-rigid image registration due to its linear computational complexity and ease of implementation over the last few decades. Optical flow registration using variational minimization and various similarity measures is well studied in the context of computer vision [7,8,9,10].

In the last two decades, variational methods have played an important role in the area of optical flow estimation. Starting from the original approaches of Horn and Schunck [11] as well as Lucas and Kanade [12], a wide range of variational optical flow methods have been developed to deal with the shortcomings of previous models during recent years. Brox et al. [7] studied an effective variational approach for optical flow computations using a coarse-to-fine strategy, named the warping technique, based on a brightness constancy, a gradient constancy assumption, and a discontinuity-preserving spatio-temporal smoothness constraint. Bruhn et al. [8] investigated the usefulness of bidirectional multigrid methods for variational optical flow computations to such an extent that they allow for real-time performance on standard hardware, and also investigated the use of decoupled and coupled versions of the classical Gauß–Seidel method as basic iterative solvers for our multigrid schemes. Woo et al. [9] proposed a non-rigid ultrasound image registration method using the intensity as well as the local phase information under a variational framework. Brox et al. [10] presented a solution to the inherent problem of current state-of-the-art optical flow estimation methods to estimate large motions of small structures by integrating correspondences from descriptor matching into a variational approach. Recently, deep learning-based methods have been applied to medical image registration and motion tracking. Convolutional neural networks (CNNs) are trained to predict optical flow [13]. Most of these works were supervised methods [14], with the need for a ground truth optical flow for training, which is nearly impossible to obtain for medical images. Unsupervised methods [15] learn the deformation field by a loss function of the similarity between the fixed image and the target image. Ye et al. [13] proposed a novel bi-directional unsupervised diffeomorphic registration network to track regional myocardium motion on t-MRI images. However, few papers use deep learning-based methods to improve optical flow registration for medical images, especially ultrasound images. Although efforts have been made to seek more accurate regularization terms, optical flow approaches lack accuracy, especially for MRI or ultrasound motion tracking, due to the tag fading and large deformation problems [16].

Inspired by these works, as the inaugural phase of our research, in this paper, we propose an effective variational registration method that simultaneously integrates intensity, local phase information, descriptor matching, and the warping technique [17,18]. The local phase information, which is invariant to image brightness, contrast, and noise, can provide more local structural information than the intensity feature, so it is well-suited for ultrasound image registration [19]. A coarse-to-fine optimization strategy named the warping technique implements the non-linearized optical flow constraint used in [20,21]. The constraints in the variational model successively remove mismatches and provide accuracy, while descriptor matching can guide the solution towards large displacements of small and independently moving structures using this technique [10]. As far as we know, this is an effective effort in simultaneously solving the ultrasound image registration problem using intensity, local phase information, descriptor matching, and warping technique under the variational framework. Notably, the descriptor-matching and warping technique has been utilized for the first time within the framework of optical flow non-rigid registration for ultrasound images. This establishes the groundwork for integrating deep learning-based approaches into the framework of optical flow non-rigid registration for ultrasound images in the subsequent research phase.

Our paper is organized as follows. We give a short review of the variational optical flow methods in Section 1. The local phase information and its properties are introduced. The registration method derived under a variational framework is presented, and the theoretical foundation of the warping technique as a numerical approximation step is given in Section 2. Experimental results using simulation and in vivo ultrasound images are shown in Section 3. Finally, concluding remarks and future research directions are given in Section 4.

## 2. Materials and Methods

This retrospective study was approved by the institutional review board of First Affiliated Hospital of Kunming Medical University. The requirement for informed consent was waived.

The problem of ultrasound image registration is equivalent to finding the displacement vector under diffeomorphism between the source and the target images. The displacement vector denotes the motion field of an image pixel.

### 2.1. Local Phase Information

The local phase information provides a qualitative, contrast invariant, local structural description of an image, which can be derived from the monogenic signal [19,22,23] and the Difference in Gaussian (DoG) function [24,25] by using the Riesz transformation.

The monogenic signal may be computed from the output of three filters. First, a rotationally symmetric, zero-mean filter is applied to the image to give a bandpass image ***I***_b_: this constitutes the even component of the signal. The odd component is composed of the response of two anti-symmetric filters to the even part [26]. These two filters, ***h***_1_ and ***h***_2_, are described in the Fourier domain by:

(1) $$H_{1}\left(u_{1},u_{2}\right)=\frac{u_{1}}{\sqrt{{u_{1}}^{2}+{u_{2}}^{2}}}\;\mathrm{and}\;H_{2}\left(u_{1},u_{2}\right)=\frac{u_{2}}{\sqrt{{u_{1}}^{2}+{u_{2}}^{2}}}$$

where ***u***_1_, ***v***_1_ are frequency-domain variables. The local phase ***ϕ*** and local energy ***E*** can then be calculated from these filter responses and the band-pass image ***I***_b_ as follows:

(2) $$\phi \left(x,y\right)=\tan^{-1}\left(\frac{I_{b}}{\sqrt{\left(h_{1}*I_{b}\right)+\left(h_{2}*I_{b}\right)}}\right)$$

(3) $$E\left(x,y\right)=\sqrt{I^{2}+{\left(h_{1}*I_{b}\right)}^{2}+{\left(h_{2}*I_{b}\right)}^{2}}$$

### 2.2. Basic Assumption

Before deriving a variational formulation for the registration approach, we give an intuitive idea of which constraints should be included in such a model.

* Grey value constancy assumption.

It has been assumed that the grey value of a pixel is not changed by the displacement since optical flow estimation was put forward.

(4) $$I\left(x,y,t\right)=I\left(x+u,y+v,t+1\right)$$

where $I:\Omega \subset R^{3}\to R$ denotes an image sequence, and (***u***, ***v***) is the local displacement vector between an image at time t and another image at time ***t*** + 1.

* Gradient constancy assumption.

The grey value constancy assumption is quite susceptible to slight changes when there are small variations in the grey value. The gradient criterion of the grey value is invariant under grey value changes to determine the displacement vector, and can be useful to prevent linearization. The gradient criterion can also be assumed not to vary due to the displacement [25], which gives:

(5) $$\nabla I\left(x,y,t\right)=\nabla I\left(x+u,y+v,t+1\right)$$

here ***∇*** = (∂***x***, ∂***y***) T denotes the spatial gradient. The linearized version of the grey value constancy assumption yields the famous optical flow constraint [11]:

(6) $$I_{x}u+I_{y}v+I_{t}=0$$

where ***I_x_*** and ***I_y_*** denote partial derivatives ∂***_x_ I*** and ∂***_y_ I***. However, this linearization is only valid under the assumption that the image changes linearly along the displacement, which is in general not the case, especially for large displacements.

* Local phase information and its gradient constancy assumption.

Equation (6) alone does not carry sufficient and accurate information for the registration due to the low contrast and low-resolution nature of the ultrasound images, while the local phase value is a more accurate feature than the intensity value. It is assumed that the local phase value and its gradient value are also similar along their temporal trajectory curves:

(7) $$LP\left(x,y,t\right)=LP\left(x+u,y+v,t+1\right)$$

(8) $$\nabla LP\left(x,y,t\right)=\nabla LP\left(x+u,y+v,t+1\right)$$

where ***LP*** is the local phase of a given image, and ∇***LP*** denotes its spatial gradient.

* Smoothness assumption and multiscale approach.

It is assumed that the smoothness of the flow field can either be applied solely to the spatial domain or to the spatio-temporal domain. And it is sensible to generalize the smoothness assumption by demanding a piecewise, smooth-flow field. In order to find the global minimum, applying a multiresolution strategy is so important that it is more efficient to downsample the images based on the sampling theorem [7].

### 2.3. The Variational Model

Non-rigid registration schemes are formulated as an optimization procedure that maximizes a similarity criterion between the source image and the target image, to find the optimal transformation. Transformation models could be parametric models, including B-spline free-form deformation, and non-parametric models, including the variational method and the demons method [27].

Marloes et al. [28] showed that in non-rigid registration of intraoperatively acquired 3D ultrasound data of brain tumours, the accuracy of the B-spline method is basically the same as that of the traditional optical flow method. Durghalli et al. [29] showed that in most conventional implementations, the original Horn and Schunck optical flow algorithm is more practical and accurate than the Demons algorithm for doing deformable image registration on thoracic 4DCT images. Zikic et al. [30] showed that there is a striking similarity between the parametrizations of the B-spline free-form approach and the demons approach.

In ultrasound image registration [28,29,30,31], if stability, robust local control capability, and rapid computational speed are prioritized, the B-spline method proves to be superior; however, it is constrained by limited detail capture capability, low resolution, and the presence of local extremum issues. When simplicity of implementation, rapid computational speed, and relative robustness against speckle noise are sought, the demons method is preferable, albeit with limitations in handling large deformations, insufficient smoothness of the motion field, and susceptibility to inconsistencies in image grayscale. For scenarios demanding rich motion details, high registration accuracy, smooth motion fields, and strong physical interpretability, the variational optical flow method is optimal, but it has high computational complexity, relatively slow solution speed, complex parameter tuning, and sensitivity to noise.

Based on the above investigation and analysis, in order to capture small motion details and analyze the dynamic behaviour of carotid artery ultrasound images of human subjects, we adopt a variational optical flow model and overcome these difficulties, such as relatively slow solution speed, complex parameter tuning, and sensitivity to noise. With these descriptions above, it is straightforward to derive an energy functional that penalizes deviations from these model assumptions.

Let ***x*** = (***x***,***y***) ^T^ and ***w***(***x***) = ***w*** (***x***,***y***) = (***u*** (***x***,***y***), ***v*** (***x***,***y***)) ^T^. Then a common assumption is that corresponding points should have the same grey value. This can be expressed by the energy:

(9) $$E_{gray}\left(w\right)=\int_{\Omega }\Psi \left({\left|I_{2}\left(x+w\left(x\right)\right)-I_{1}\left(x\right)\right|}^{2}\right)dx$$

where ***Ψ*** is a quadrature penalizer [17,18,32] (e.g., $\Psi \left(s^{2}\right)=\sqrt{s^{2}+\varepsilon^{2}}$ which results in a modified ***L***^1^ norm, and where ***ε*** is a small number), and ***I***_1_ and ***I***_2_ are the intensity of the source image and target image, respectively.

Due to illumination effects, matching the grey value is not always reliable. Therefore, we use the gradient of local phase value to supplement the constraint in (14), which is invariant to additive brightness changes [33]:

(10) $$E_{grad}\left(w\right)=\int_{\Omega }\Psi \left({\left|\nabla LP_{2}\left(x+w\left(x\right)\right)-\nabla LP_{1}\left(x\right)\right|}^{2}\right)dx$$

with the same function for ***Ψ*** as above, and ∇***LP***_1_, ∇***LP***_2_ are the gradient values of the local phase of the source image and target image, respectively.

The smoothness term is derived under the assumption of a piecewise smooth flow field. This is achieved by penalizing the total variation in the flow field [34,35], which can be expressed as follows:

(11) $$E_{smooth}\left(w\right)=\int_{\Omega }\Psi \left({\left|\nabla u\left(x\right)\right|}^{2}+{\left|\nabla v\left(x\right)\right|}^{2}\right)dx$$

with the same function for ***Ψ*** as above.

In order to enforce a smooth flow field and provide subpixel accuracy, we combine descriptor matching with the variational model and its coarse-to-fine optimization. The point correspondences [36] from descriptor matching are integrated into the variational approach by adding match term:

(12) $$E_{match}\left(w\right)=\int \delta \left(x\right)\rho \left(x\right)\Psi \left({\left|w\left(x\right)-w_{1}\left(x\right)\right|}^{2}\right)dx$$

where ***w***_1_(***x***) denotes the correspondence vectors obtained by descriptor matching at some points ***x***. The same function for ***Ψ*** is as above. ***δ***(***x***) is 1 if there is a descriptor available in frame 1 at point ***x***; otherwise, it is 0. Each correspondence is weighted by its matching score ***ρ***(***x***), which is defined as follows:

(13) $$\rho (x_{i})=\frac{d_{2}-d_{1}}{d_{1}}$$

where ***d***_1_ and ***d***_2_ denote the distances of the best and the second-best match, respectively. The distances are the sums of squared differences in warped patches [37]. Equation (12) assumes that the descriptors are already matched. We can formulate this matching task as another energy term that is called the descriptor term to be minimized:

(14) $$E_{\mathrm{desc}}\left(w_{1}\right)=\int \delta \left(x\right){\left|f_{2}\left(x+w_{1}\left(x\right)\right)-f_{1}\left(x\right)\right|}^{2}dx$$

where ***f***_1_(***x***) and ***f***_2_(***x***) denote the sparse fields of feature vectors in frame 1 and frame 2, respectively. The auxiliary variable ***w***_1_ allows for integrating discrete descriptor matching into a continuous approach in the form of soft constraints. With this auxiliary variable and the coupling term Ematch, discrete matching would be compatible with the variational setting [10,38].

The total energy functional can be expressed as the weighted sum of the grey term, gradient term, smoothness term, match term, and descriptor term:

(15) $$E\left(w\right)=E_{gray}\left(w\right)+\gamma E_{grad}\left(w\right)+\alpha E_{smooth}\left(w\right)+\beta E_{match}\left(w,w_{1}\right)+E_{desc}\left(w_{1}\right)$$

where ***α***, ***β***, and ***γ*** are tuning parameters which steer the importance of smoothness, region correspondences, and gradient constancy, respectively. We can adjust the importance of the smoothness term and the other term by tuning parameter ***α***. We can adjust the importance of the match term and the other terms by choosing ***β***. Likewise, we can adjust the importance of the gradient term and the other terms by choosing ***γ***.

The paper [10] demonstrates that integrating the correspondences from descriptor matching into a variational optical flow mode exhibits considerable robustness to minor variations in the tuning parameters. The experiments proposed in the paper [39] show results of an experiment on parameters (***α***, ***β***, and ***γ***) variation, and the results are quite stable. More significantly, utilizing a fixed set of parameters, this approach is capable of generating reasonable flow estimates for a variety of sequences.

Because ***α***, ***β***, and ***γ*** can be determined manually according to qualitative evidence on a large variety of videos, or be estimated automatically from ground truth data, we conduct our parameter comparison experiments and heuristically find the parameter values (***α*** = 30, ***β*** = 300, and ***γ*** = 80) that minimize the energy functional the most. The effects are significant. So, we fixed all parameters at ***α*** = 30, ***β*** = 300, and ***γ*** = 80. It is worth noting that this set of parameters optimized for ultrasound images puts more emphasis on smoothness than the parameters optimized for the Middlebury data used in the paper [10]. In the subsequent research phase, these parameters can be determined through deep learning-based approaches within the framework of optical flow non-rigid registration for ultrasound images.

### 2.4. Minimization

The final goal is to find a minimum equal to or similar to the global minimum of the energy in Equation (15). Because this function is highly non-convex, we need reasonable approximation schemes that find a good initial guess of the solution.

We combine the descriptor-matching method and a continuation method that produces initial guesses. Both methods are complementary in the way they simplify the energy, which can be globally optimized. Descriptor matching neglects regularity, whereas the continuation method neglects image details. We start with descriptor matching and explain the continuation method afterwards [40].

A. Descriptor matching

The descriptor matching part focuses on minimizing ***E***_desc_(***w***_1_) independently from the rest of the energy. Decoupling ***E***_desc_(***w***_1_) enables global optimization of this subproblem.

Let ***δ***(***x***) define a discrete grid in frame 1 and ***δ***′(***x***) another grid in frame 2. Usually, ***δ***′(***x***) will be a finer grid than ***δ***(***x***). Equation (14) can be expressed as follows:

(16) $$E_{desc}\left(w_{1}\right)=\sum_{i,\delta (x_{i})=1}{\left|f_{2}\left(x_{i}+w_{1}\left(x_{i}\right)\right)-f_{1}\left(x_{i}\right)\right|}^{2}$$

Thus, we can optimize w_1_ at each grid point xi independently. This can be achieved by evaluating the energy for all possible grid points x_j_ of ***δ***′(***x***) and choosing ***x***_j_ for which this energy is minimal. The optimal ***w***_1_(***x***_i_) = ***x***_j_ − ***x***_i_. It can be reduced by using efficient nearest-neighbour search [41].

We have investigated here three different methods: one based on region matching proposed in [37], one based on histograms of oriented gradients (HOG) descriptors [42], and one based on geometric blur (GB) [43]. Among the descriptor-matching techniques, the HOG descriptor leads to the smallest loss in accuracy and produces the fewest mismatches, followed by GB and region matching. The computation of the HOG descriptors is also the most efficient one. When analyzing more frames, this qualitative behaviour persists: HOG descriptors produce the fewest mismatches, whereas GB descriptors tend to capture more details. There are also possibilities to combine both descriptors. However, we did not further investigate this in detail.

HOG is a type of feature descriptor that defines the entire image with a few pixel points of representation. HOG focuses on the shape of the region of interest that clearly describes the edges of images with gradient and orientation [44]. Speckle noise of ultrasound images mainly reduces the levels of contrast with changes in the pixels, so speckle-reducing anisotropic diffusion (SRAD) filtering [45] is first performed on these ultrasound images as a pre-processing step since the intensity and its gradient can be sensitive to noise, resulting in undesired matching. The SRAD algorithm is capable of not only suppressing speckle noise but also retaining the texture details and reinforcing the edges of the image. Owing to the reduction in speckle noise, HOG can more effectively fulfil its function, and the variational optical flow method can be more accurate.

Because the optimization of this part is extremely simple, we only need to define reasonable descriptors and grids so that the descriptors are unique enough to limit the number of false matches, and that the grid is fine enough to capture the motion of smaller structures. We choose HOG as the descriptors for matching and define the grid δ(x) by picking a descriptor at every fourth pixel in the x- and y-direction. This reduces the matching effort by a factor of 16 compared to sampling a descriptor at every pixel. Each gradient histogram comprises 8 different orientations and is computed in a 4 × 4 neighbourhood. We apply a Gaussian kernel with ***σ*** = 0.8 in the orientation direction to reduce quantization effects.

B. Euler–Lagrange Optimization

After decoupling ***E***_desc_(***w***_1_), the remainder of Equation (15) can be minimized using the Euler–Lagrange method [7]. The additional term Ematch is convex in w and does not cause any trouble. The idea of the method is to split the original problem into a sequence of subproblems at different resolution levels by smoothing the input images. The subproblem in each continuation step is convex and can be globally optimized for fixed correspondences ***w***_1_. The proposed total energy functional satisfies the following Euler–Lagrange equation with respect to u and v as follows:

$$\begin{array}{l} \Psi^{\prime }\left(I_{z}^{2}\right)I_{z}I_{x}+\gamma \Psi^{\prime }\left(LP_{xz}^{2}+LP_{yz}^{2}\right)\left(LP_{xx}LP_{xz}+LP_{xy}LP_{yz}\right)\\ +\beta \rho \Psi^{\prime }\left({\left(u-u_{1}\right)}^{2}+{\left(v-v_{1}\right)}^{2}\right)\left(u-u_{1}\right)-\alpha div\left(\Psi^{\prime }\left({\left|\nabla u\right|}^{2}+{\left|\nabla v\right|}^{2}\right)\nabla u\right)=0 \end{array}$$

(17) $$\begin{array}{l} \Psi^{\prime }\left(I_{z}^{2}\right)I_{z}I_{y}+\gamma \Psi^{\prime }\left(LP_{xz}^{2}+LP_{yz}^{2}\right)\left(LP_{xy}LP_{xz}+LP_{yy}LP_{yz}\right)\\ +\beta \rho \Psi^{\prime }\left({\left(u-u_{1}\right)}^{2}+{\left(v-v_{1}\right)}^{2}\right)\left(v-v_{1}\right)-\alpha div\left(\Psi^{\prime }\left({\left|\nabla u\right|}^{2}+{\left|\nabla v\right|}^{2}\right)\nabla v\right)=0 \end{array}$$

where:

***I***_x_ = ∂_x_***I***_2_(***x*** + ***w***), ***I***_y_ = ∂_y_***I***_2_(***x*** + ***w***), ***I***_z_ = ***I***_2_(***x*** + ***w***) − ***I***_1_(***x***)

***LP***_x_ = ∂_x_***LP***_2_(***x*** + ***w***), ***LP***_y_ = ∂_y_***LP***_2_(***x*** + ***w***), ***LP***_xy_ = ∂_xy_***LP***_2_(***x*** + ***w***)

***LP***_xx_ = ∂_xx_***LP***_2_(***x*** + ***w***), ***LP***_yy_ = ∂_yy_***LP***_2_(***x*** + ***w***) (18)

***LP***_z_= ***LP***_2_(***x*** + ***w***) − ***LP***_1_(x), ***LP***_xz_ = ∂_x_***LP***_z_, ***LP***_yz_ = ∂_y_***LP***_z_

and $\Psi^{\prime }\left(x\right)=\frac{1}{2\sqrt{x+\varepsilon^{2}}}$ where ***ε*** is a small number. In our simulation and experiments, we choose ***ε*** = 0.001.

C. Numerical Approximation

The use of fixed-point iterations on w can be used to resolve the nonlinearity in their argument ***w*** = (***u***, ***v***, 1)^T^ of the preceding Euler–Lagrange Equation (17). These fixed-point iterations are combined with a downsampling strategy to better approximate the global optimum of the energy. We use a very fine pyramid, where the image at level k is a downsampled version of the input image with a downsampling factor of 0.95^(kmax−k)^ to allow for smooth transitions between levels. k_max_ is chosen such that discrete derivative filters can still be applied. Moreover, the full pyramid of images is used, starting with the smallest possible image at the coarsest grid. Let ***w***_k_ = (***u***_k_, ***v***_k_, 1)^T^, ***k*** = 0, 1, …, with the initialization ***w***_0_ = (0, 0, 1)^T^ at the coarsest grid. Further, let $I_{*}^{k}$ and $LP_{*}^{k}$ be the abbreviations defined in Equation (18) but with the iteration variable ***w***^k^ instead of ***w***. Then ***w***^k+1^ will be the solution of:

(19) $$\begin{array}{l} \Psi^{\prime }{\left(I_{z}^{k+1}\right)}^{2}I_{x}^{k}I_{z}^{k+1}+\gamma \Psi^{\prime }\left({\left(LP_{xz}^{k+1}\right)}^{2}+{\left(LP_{yz}^{k+1}\right)}^{2}\right)\left(LP_{xx}^{k}LP_{xz}^{k+1}+LP_{xy}^{k}LP_{yz}^{k+1}\right)\\ +\beta \rho \Psi^{\prime }\left({\left(u^{k+1}-u_{1}\right)}^{2}+{\left(v^{k+1}-v_{1}\right)}^{2}\right)\left(u^{k+1}-u_{1}\right)\\ -\alpha div\left(\Psi^{\prime }\left({\left|\nabla u^{k+1}\right|}^{2}+{\left|\nabla v^{k+1}\right|}^{2}\right)\nabla u^{k+1}\right)=0 \end{array}$$

$$\begin{array}{l} \Psi^{\prime }{\left(I_{z}^{k+1}\right)}^{2}I_{y}^{k}I_{z}^{k+1}+\gamma \Psi^{\prime }\left({\left(LP_{xz}^{k+1}\right)}^{2}+{\left(LP_{yz}^{k+1}\right)}^{2}\right)\left(LP_{xy}^{k}LP_{xz}^{k+1}+LP_{yy}^{k}LP_{yz}^{k+1}\right)\\ +\beta \rho \Psi^{\prime }\left({\left(u^{k+1}-u_{1}\right)}^{2}+{\left(v^{k+1}-v_{1}\right)}^{2}\right)\left(v^{k+1}-v_{1}\right)\\ -\alpha div\left(\Psi^{\prime }\left({\left|\nabla u^{k+1}\right|}^{2}+{\left|\nabla v^{k+1}\right|}^{2}\right)\nabla v^{k+1}\right)=0 \end{array}$$

As soon as a fixed point in wk is reached, we change to the next finer scale and use this solution as initialization for the fixed-point iteration on this scale. This new system is still nonlinear because of the nonlinear function Ψ and the symbols $I_{*}^{k+1}$ and $LP_{*}^{k+1}$. In order to remove the nonlinearity in $I_{*}^{k+1}$ and $LP_{*}^{k+1}$, first-order Taylor expansions are used:

$$I_{z}^{k+1}=I_{z}^{k}+I_{x}^{k}du^{k}+I_{y}^{k}dv^{k},$$

(20) $$LP_{xz}^{k+1}=LP_{xz}^{k}+LP_{xx}^{k}du^{k}+LP_{xy}^{k}dv^{k},$$

$$LP_{yz}^{k+1}=LP_{yz}^{k}+LP_{xy}^{k}du^{k}+LP_{yy}^{k}dv^{k},$$

Let $u^{k+1}=u^{k}+du^{k}$ and $v^{k+1}=v^{k}+dv^{k}$, and for a fixed ***k***, we split the unknowns ***u***^k+1^, ***v***^k+1^ in the solutions of the previous iteration step ***u***^k^ and ***v***^k^, and unknown increment du^k^ and d***v***^k^. For better readability, let:

$${\left(\Psi_{1}^{\prime }\right)}^{k}=\Psi^{\prime }\left({\left(I_{z}^{k}+I_{x}^{k}du^{k}+I_{y}^{k}dv^{k}\right)}^{2}\right)$$

(21) $${\left(\Psi_{2}^{\prime }\right)}^{k}=\Psi^{\prime }\left({\left(LP_{xz}^{k}+LP_{xx}^{k}du^{k}+LP_{xy}^{k}dv^{k}\right)}^{2}+{\left(LP_{yz}^{k}+LP_{xy}^{k}du^{k}+LP_{yy}^{k}dv^{k}\right)}^{2}\right)$$

$${\left(\Psi_{3}^{\prime }\right)}^{k}=\Psi^{\prime }\left({\left(u^{k}+du^{k}-u_{1}\right)}^{2}+{\left(v^{k}+dv^{k}-v_{1}\right)}^{2}\right)$$

$${\left(\Psi_{4}^{\prime }\right)}^{k}=\Psi^{\prime }\left({\left|\nabla \left(u^{k}+du^{k}\right)\right|}^{2}+{\left|\nabla \left(v^{k}+dv^{k}\right)\right|}^{2}\right)$$

Then let d***u***^k,0^ = 0 and d***v***^k,0^ = 0 be our initialization, and let d***u***^k,l^ and d***v***^k,l^ denote the iteration variables at some step l. Finally, with Equation (21), the linear system of equations in d***u***^k, l+1^, d***v***^k, l+1^ reads:

(22) $$\begin{array}{l} {\left(\Psi_{1}^{\prime }\right)}^{k,l}I_{x}^{k}\left(I_{z}^{k}+I_{x}^{k}du^{k,l+1}+I_{y}^{k}dv^{k,l+1}\right)+\gamma {\left(\Psi_{2}^{\prime }\right)}^{k,l}LP_{xx}^{k}\left(LP_{xz}^{k}+LP_{xx}^{k}du^{k,l+1}+LP_{xy}^{k}dv^{k,l+1}\right)\\ +\gamma {\left(\Psi_{2}^{\prime }\right)}^{k,l}LP_{xy}^{k}\left(LP_{yz}^{k}+LP_{xy}^{k}du^{k,l+1}+LP_{yy}^{k}dv^{k,l+1}\right)+\beta \rho {\left(\Psi_{3}^{\prime }\right)}^{k,l}\left(u^{k}+du^{k,l+1}-u_{1}\right)\\ -\alpha div\left({\left(\Psi_{4}^{\prime }\right)}^{k,l}\nabla \left(u^{k}+du^{k,l+1}\right)\right)=0 \end{array}$$

$$\begin{array}{l} {\left(\Psi_{1}^{\prime }\right)}^{k,l}I_{y}^{k}\left(I_{z}^{k}+I_{x}^{k}du^{k,l+1}+I_{y}^{k}dv^{k,l+1}\right)+\gamma {\left(\Psi_{2}^{\prime }\right)}^{k,l}LP_{xy}^{k}\left(LP_{xz}^{k}+LP_{xx}^{k}du^{k,l+1}+LP_{xy}^{k}dv^{k,l+1}\right)\\ +\gamma {\left(\Psi_{2}^{\prime }\right)}^{k,l}LP_{yy}^{k}\left(LP_{yz}^{k}+LP_{xy}^{k}du^{k,l+1}+LP_{yy}^{k}dv^{k,l+1}\right)+\beta \rho {\left(\Psi_{3}^{\prime }\right)}^{k,l}\left(v^{k}+dv^{k,l+1}-v_{1}\right)\\ -\alpha div\left({\left(\Psi_{4}^{\prime }\right)}^{k,l}\nabla \left(v^{k}+dv^{k,l+1}\right)\right)=0 \end{array}$$

The coarse-to-fine warping technique can be theoretically justified as a numerical approximation or a single minimization problem [17,46,47,48]. Image registration techniques relying on non-linearized constancy assumptions have access to an efficient multiresolution approach for minimizing their energy functionals. We use this technique to solve Equation (22).

## 3. Results

### 3.1. Simulation Model

In this study, the ultrasound simulation is conducted with the Field II software (version 3.24), which was created by Jensen [37,44]. Based on linear systems theory, this software simulates an ultrasound field using the spatial impulse response method proposed by Tupholme and Stepanishen [45,46]. Since any transducer can be simulated by splitting the aperture into some kind of small, shaped subapertures, and any transducer excitation and apodization can be included in the calculation, it is feasible to obtain realistic simulated ultrasound images.

**Figure 1:**
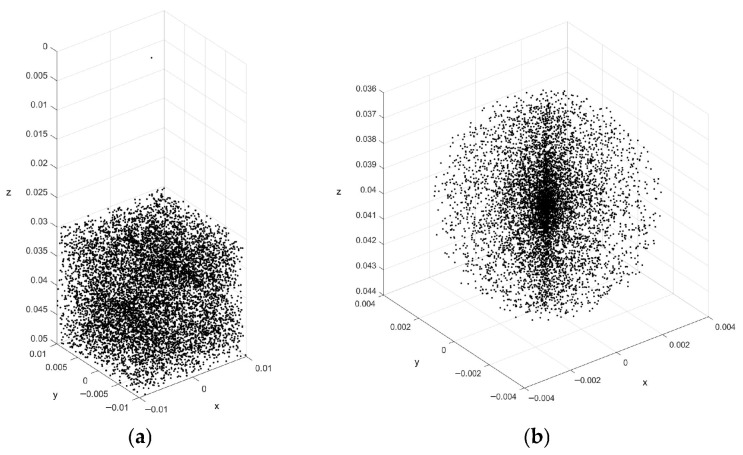
(**a**) The scatter model of the cyst phantom and (**b**) the scatter model of the spheroid cyst region.

We constructed a spherical cyst expansion phantom whose point scatters are randomly placed and have a scattering amplitude with a Gaussian distribution. In the simulation, the cyst phantom consists of a spheroid cyst region and a homogeneous tissue region, which typically consists of 100,000 scatters. The spheroid cyst region is a sphere with a radius of 3 mm in the centre of the cyst phantom, and the rest of the cyst phantom is the tissue region. The scatters in the phantom are generated by finding their random position within a 20 × 20 × 20 mm^3^ cube, and then ascribing a Gaussian distributed amplitude to each scatter. If the scatter resides within a spheroid cyst region, the amplitude is set to zero. The scatter models of the cyst phantom and the spheroid cyst region are shown in Figure 1a,b. Other parameters are set as: the centre frequency of 12 MHz, the sampling frequency of 100 MHz, physical and active elements of 512 and 64. By using the established phantom with preset parameter values, the ultrasound RF signals and images are simulated using Field II.

**Figure 2:**
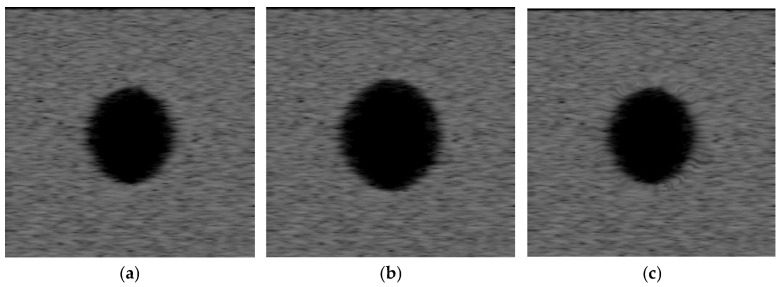
(**a**) The source image, (**b**) the target image (the radius of the spheroid cyst region is 3 mm), and (**c**) the registered image obtained by the proposed algorithm.

**Figure 3:**
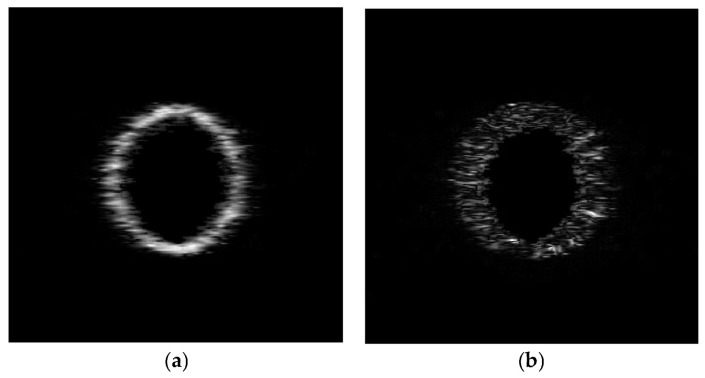
(**a**) The difference image between the source and the target images, and (**b**) the difference image between the source and the registered image.

**Figure 4:**
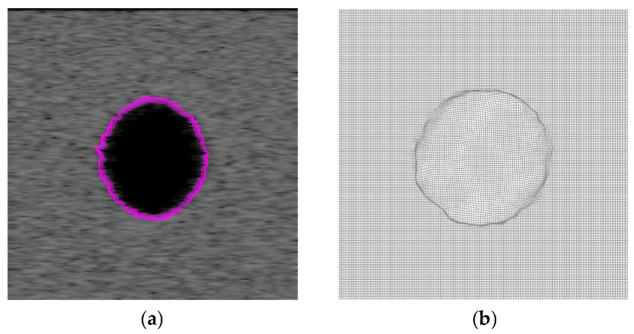
(**a**) The displacement vector from the cyst boundary in Figure 2a (red arrows), and (**b**) the deformation field.

Then we increase the radius of the cyst spheroid from 3 mm to 3.6 mm, and the volume of the tissue region decreases. The displacement vectors that incur deformations of ultrasound images are generated by increasing the radius of the spheroid cyst. The proposed algorithm is used to evaluate registration accuracy under nonrigid deformations of the spherical cyst expansion phantom. For visual evaluation of the proposed algorithm, the source and target ultrasound images are shown in Figure 2a,b, respectively, along with their intensity difference in Figure 3a. The registered ultrasound image is as shown in Figure 2c, and the intensity difference between the source and the registered ultrasound images is as shown in Figure 3b. The displacement vector from the source image is presented in Figure 4a. The deformation field is presented in Figure 4b.

Experimental results with the synthetic example are shown in Table 1, where ***v*** and ***v’*** denote the ground truth displacement vector and the computed one using different methods, respectively. The absolute intensity differences (AD_v-v’_), the mutual information (MI), and the mean of the sum of squared intensity differences (SSD) between target and registered images are used as quantitative performance metrics. Three models using different features are compared in Table 1. Firstly, we use the intensity term and gradient term [15], namely:

$$\int_{\Omega }\Psi \left({\left|I_{2}\left(x+w\left(x\right)\right)-I_{1}\left(x\right)\right|}^{2}+\gamma {\left|\nabla I_{2}\left(x+w\left(x\right)\right)-\nabla I_{1}\left(x\right)\right|}^{2}\right)dx$$

which is denoted by I + G in Table 1. Next, we use the intensity term, gradient term, match term and descriptor term, namely:

$$\int_{\Omega }\Psi \left({\left|I_{2}\left(x+w\left(x\right)\right)-I_{1}\left(x\right)\right|}^{2}+\gamma {\left|\nabla I_{2}\left(x+w\left(x\right)\right)-\nabla I_{1}\left(x\right)\right|}^{2}\right)dx+\beta E_{match}\left(w,w_{1}\right)+E_{desc}\left(w_{1}\right)$$

which is denoted by I + G + D in Table 1. The intensity term and gradient term of the local phase are used in the third one, namely:

$$\int_{\Omega }\Psi \left({\left|I_{2}\left(x+w\left(x\right)\right)-I_{1}\left(x\right)\right|}^{2}+\gamma {\left|\nabla LP_{2}\left(x+w\left(x\right)\right)-\nabla LP_{1}\left(x\right)\right|}^{2}\right)dx$$

which is denoted by I + LP in Table 1. The last one is the proposed model that uses the intensity term, gradient term of local phase, match term and descriptor term, namely:

$$\int_{\Omega }\Psi \left({\left|I_{2}\left(x+w\left(x\right)\right)-I_{1}\left(x\right)\right|}^{2}+\gamma {\left|\nabla LP_{2}\left(x+w\left(x\right)\right)-\nabla LP_{1}\left(x\right)\right|}^{2}\right)dx+\beta E_{match}\left(w,w_{1}\right)+E_{desc}\left(w_{1}\right)$$

which is denoted by I + LP +D in Table 1.

In our simulation, we choose ***σ*** = 0.8, ***α*** = 30, ***β*** = 300, and ***γ*** = 80. As to the smoothness term, the first three models adopt the same one as the proposed model. We see from Table 1 that the proposed model has a smaller registration error in terms of the absolute error as well as smaller SSD, and has a higher MI than the other three models.

### 3.2. In Vivo Image

**Figure 5:**
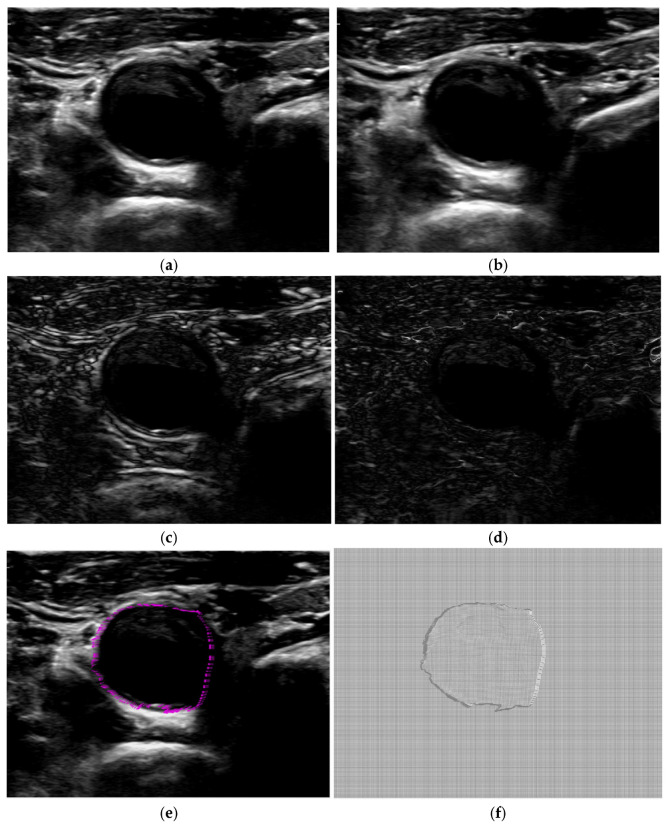
In vivo carotid artery cross-section ultrasound images: (**a**) the source image, (**b**) the target image, (**c**) the difference image between the source and target image, (**d**) the results obtained by the proposed algorithm, (**e**) the displacement vector from the boundary in a (red arrows), and (**f**) the deformation field.

**Figure 6:**
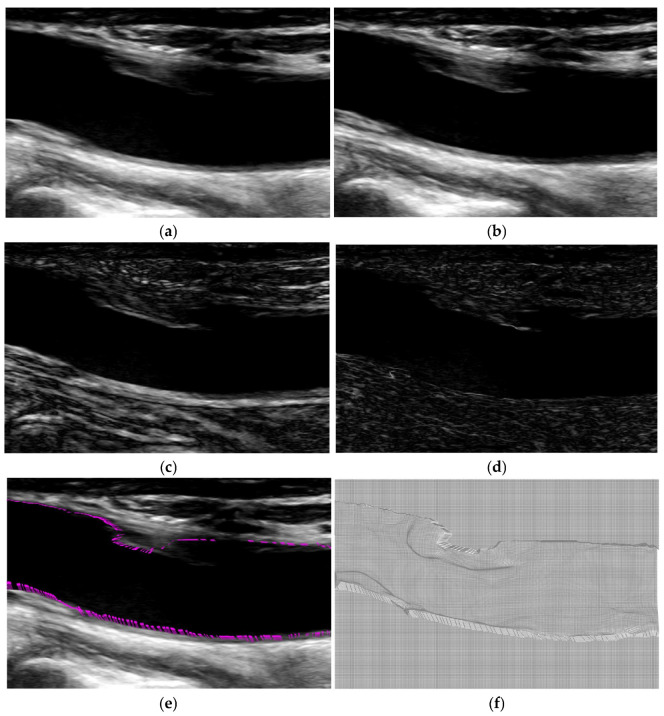
In vivo local carotid artery ultrasound images: (**a**) the source image, (**b**) the target image, (**c**) the difference image between the source and target image, (**d**) the results obtained by the proposed algorithm, (**e**) the displacement vector from the boundary in a (red arrows), and (**f**) the deformation field.

Furthermore, we use in vivo carotid artery ultrasound images of human subjects for performance comparison. The proposed algorithm is used to register vivo carotid artery ultrasound images. The source and the target ultrasound images are the previous and the current image frames of the carotid artery cross-section, and the local carotid artery when the heart beats. The processed source and the target ultrasound images of the carotid artery cross-section and local carotid artery are shown in Figure 5 and Figure 6a,b, along with their intensity difference in Figure 5 and Figure 6c. For visual evaluation of the proposed algorithm, the difference image between the target and the registered images is presented in Figure 5 and Figure 6d. The displacement vectors from the source image are presented in Figure 5 and Figure 6e. The deformation fields are presented in Figure 5 and Figure 6f. As shown in the obtained deformation fields, the proposed method is able to find the displacement of pixels in the dark region that have similar intensity levels.

A variety of similarity metrics used for ultrasound registration are discussed in the paper [31], including mutual information (MI), normalized correlation coefficient (NCC), Correlation Coefficient (CC), Sum of Squared Differences (SSD), and so on. Since there is no ground truth in the in vivo data, in situations where manual landmark correspondences are not available, surrogate measures are used to evaluate accuracy. The most straightforward measures of this kind include absolute intensity differences (AD), the root-mean-square intensity difference (RMSD), and the mean of the sum of squared intensity differences (SSD) between the target image and the registered image. Other similarity measures, such as MI, NCC, CC, and structural similarity index (SSIM), can also be used in [13,49,50]. For mono-modality medical image registration, these standard pixel-based metrics are widely adopted medical image similarity measures.

MI is a popular image similarity metric for both rigid and non-rigid medical image registration. MI seeks a transform that aligns two (ultrasound) images or volumes by maximizing their mutual information. The MI metric measures how much information one variable (image or volume) contains about the other. MI is robust to outliers, and it is efficient to use in optimization, making MI an excellent metric. NCC is another widely used similarity metric. NCC calculates the correlation between two functions, and it is considered to work best with monomodal registration between two images acquired with the same characteristic curves. The NCC metric has been incorporated in both rigid and non-rigid registration algorithms with good results. SSD is perhaps the simplest standard similarity metric for rigid and non-rigid image registration. This metric calculates the sum of squared differences in pixels’ or voxels’ intensity from both reference and moving images or volumes. As this metric requires both reference and moving images/volumes to have the same intensity range, SSD is best-suited for mono-modality ultrasound registration [31]. All of these similarity metrics are iteratively optimized, which is time-consuming.

We compare registration accuracy using various similarity measures, such as the mean SSD, the MI, and the NCC, in Table 2 and Table 3. Since we used the L1 norm, which is similar to the mean SSD, comparison using more generic metrics such as MI and NC demonstrates the validity of the mean SSD as a similarity measure in ultrasound image registration (Table 2 and Table 3).

In our experiments, we choose ***σ*** = 0.8, ***α*** = 30, ***β*** = 300, and ***γ*** = 80. In order to evaluate the overall performance of different methods, the I + G model, the I + G + D model, the I + LP model, and the proposed model are applied to the same images for comparison. It can be seen that the mean SSD of the proposed algorithm is lower, while the MI and the NCC of the proposed algorithm are higher than those of the three other methods. This demonstrates that the registration results of the proposed algorithm are more accurate. The experimental results demonstrate that the proposed algorithm is efficient, quick, and robust to noise.

## 4. Discussion and Conclusions

We presented an effective non-rigid registration algorithm for ultrasound images in this paper. Specifically, the local phase information is used as a geometric feature to find correspondences of pixels in two ultrasound images that suffer from speckle, artefact, and occlusion. And the descriptor matching is used to find more image details and capture the motion of smaller structures. By combining the intensity information, the local phase information, and the descriptor matching under a variational framework, the proposed algorithm outperforms other algorithms. Different similarity measures, including AE, mean SSD, NC, and MI, were used to compare the performance of different algorithms in simulation and experiments. In the future, we would like to enhance the accuracy and efficiency of the algorithm by refining descriptor matching. Simultaneously, we would also like to use various methods, including deep learning-based approaches, to compare the capacity of different methods. The proposed algorithm can be applied to the analysis of tissue mechanical properties and object motion (e.g., beating heart) for the treatment of many diseases clinically. The proposed algorithm can be used for motion estimation of tissues and velocity estimation, or for other tracking purposes as well.

## Figures and Tables

**Table 1:** Comparison of three different similarity measures for cyst expansion phantom image registration (in units of pixels).

Method	I + G	I + G + D	I + LP	I + LP + D (Proposed)
AD_v-v’_	0.0232	0.0158	0.0132	0.0101
MI	1.5579	1.5671	1.5677	1.5789
SSD	1.6160	1.5720	1.4529	1.4470

**Table 2:** Comparison of three different similarity measures for the carotid artery cross-section image registration (in units of pixels).

Method		I + G	I + G + D	I + LP	I + LP + D (Proposed)
Carotid artery Cross-section	MI	1.1146	1.1171	1.1301	1.1321
	NCC	0.9194	0.9229	0.9567	0.9612
	SSD	13.0757	12.9381	12.7652	12.6707

**Table 3:** Comparison of three different similarity measures for the local carotid artery image registration (in units of pixels).

Method		I + G	I + G + D	I + LP	I + LP + D (Proposed)
Local carotid artery	MI	1.1896	1.1908	1.2046	1.2081
	NCC	0.9822	0.9826	0.9892	0.9897
	SSD	9.6165	9.5949	9.4666	9.2475

## Data Availability

The original contributions presented in this study are included in the article. Further inquiries can be directed to the corresponding author.
